# Effect of the Depth of Cold Water Immersion on Sleep Architecture and Recovery Among Well-Trained Male Endurance Runners

**DOI:** 10.3389/fspor.2021.659990

**Published:** 2021-03-31

**Authors:** Maxime Chauvineau, Florane Pasquier, Vincent Guyot, Anis Aloulou, Mathieu Nedelec

**Affiliations:** Laboratory of Sport, Expertise and Performance (EA 7370), French National Institute of Sport (INSEP), Paris, France

**Keywords:** polysomnography, muscle damage, core body temperature, heart rate variability, slow-wave sleep, performance, limb movements, arousals

## Abstract

**Introduction:** The aim of the present study was to investigate the effect of the depth of cold water immersion (CWI) (whole-body with head immersed and partial-body CWI) after high-intensity, intermittent running exercise on sleep architecture and recovery kinetics among well-trained runners.

**Methods:** In a randomized, counterbalanced order, 12 well-trained male endurance runners ($\dot{V}$O_2_max = 66.0 ± 3.9 ml·min^−1^·kg^−1^) performed a simulated trail (≈18:00) on a motorized treadmill followed by CWI (13.3 ± 0.2°C) for 10 min: whole-body immersion including the head (WHOLE; *n* = 12), partial-body immersion up to the iliac crest (PARTIAL; *n* = 12), and, finally, an out-of-water control condition (CONT; *n* = 10). Markers of fatigue and muscle damage—maximal voluntary isometric contraction (MVIC), countermovement jump (CMJ), plasma creatine kinase [CK], and subjective ratings—were recorded until 48 h after the simulated trail. After each condition, nocturnal core body temperature (*T*_core_) was measured, whereas sleep and heart rate variability were assessed using polysomnography.

**Results:** There was a lower *T*_core_ induced by WHOLE than CONT from the end of immersion to 80 min after the start of immersion (*p* < 0.05). Slow-wave sleep (SWS) proportion was higher (*p* < 0.05) during the first 180 min of the night in WHOLE compared with PARTIAL. WHOLE and PARTIAL induced a significant (*p* < 0.05) decrease in arousal for the duration of the night compared with CONT, while only WHOLE decreased limb movements compared with CONT (*p* < 0.01) for the duration of the night. Heart rate variability analysis showed a significant reduction (*p* < 0.05) in RMSSD, low frequency (LF), and high frequency (HF) in WHOLE compared with both PARTIAL and CONT during the first sequence of SWS. No differences between conditions were observed for any markers of fatigue and muscle damage (*p* > 0.05) throughout the 48-h recovery period.

**Conclusion:** WHOLE reduced arousal and limb movement and enhanced SWS proportion during the first part of the night, which may be particularly useful in the athlete's recovery process after exercise. Future studies are, however, required to assess whether such positive sleep outcomes may result in overall recovery optimization.

## Introduction

Elite sport requires regular competitions and training multiple times a day for consecutive days, which can make athletes' readiness for high performance challenging. An imbalance in training/competition load and recovery increases the risk of overtraining, injury, and underperformance (Kenttä and Hassmén, 1998). Several studies have assessed the interest of different strategies in accelerating the rate of recovery, with sleep and cold water immersion (CWI) demonstrating the highest scientific level of evidence (Halson, 2008; Nédélec et al., 2013).

Cold water immersion is an often-used recovery strategy (Nédélec et al., 2013), which decreases core body temperature (*T*_core_) below baseline with a peak difference occurring at 60 min post-immersion (Stephens et al., 2018). This cooling strategy is effective when repairing exercise-induced muscle damage (Ihsan et al., 2016) with larger effect for weight-bearing (running and strength training) compared with non-weight-bearing activities (Halson, 2011), which may be related to CWI-induced muscle cooling and hydrostatic pressure (Wilcock et al., 2006; Leeder et al., 2012). The main benefits of CWI are reductions in delayed onset muscle soreness, edema, and exercise-induced strength loss (Wilcock et al., 2006; Bailey et al., 2007; Leeder et al., 2012). Moreover, subjective measures of fatigue and recovery are improved in the hours and days following CWI (Wilcock et al., 2006; Halson et al., 2008). The effectiveness of CWI may be higher when a whole-body immersion is implemented compared with a partial-body protocol (Wilcock et al., 2006). A meta-analytical review has reported that whole-body immersion is significantly more effective (5.1%, *g* = 0.62) on performance recovery than partial-body immersion, i.e., immersing only the legs or arms (1.1%, *g* = 0.10; Poppendieck et al., 2013). These results can be explained by a higher reduction in *T*_core_ when the entire body is immersed (Stephens et al., 2017). In addition, the immersion of the head seems especially important to induce a maximal rate of decline in *T*_core_ (Pretorius et al., 2006). Pretorius et al. (2006) reported that head immersion in cold water (17°C for 30 min) considerably increases core body cooling rate (≈+42%) compared with when the head is not immersed.

Sleep and thermoregulation are closely related (Kräuchi and Deboer, 2010). Previous studies suggested that a maximal rate of decline in *T*_core_ close to bedtime, which occurs through an increase in cutaneous temperature and heat loss from the periphery, can favor sleep initiation and can enhance sleep propensity (Berger and Phillips, 1995; Deboer, 1998; Kräuchi and Deboer, 2010). Sleep provides a number of important psychological and physiological functions that may be fundamental to the athlete's recovery process (Nédélec et al., 2015; Walsh et al., 2020). It has notably been shown that slow-wave sleep (SWS), a component of non-rapid eye movement (NREM) sleep, is restorative and allows muscle repair and adaptation (Akerstedt and Nilsson, 2003; Dijk, 2009; Halson and Juliff, 2017). Consequently, immersing the whole body—head included—close to bedtime may be a promising CWI strategy to enhance sleep and neuromuscular recovery after a high-intensity exercise.

Some studies have examined the relationship between CWI and sleep (Robey et al., 2013; Lastella et al., 2019). Lastella et al. (2019) reported a shorter sleep onset latency after the use of CWI compared with a placebo condition among elite cyclists during a simulated hill-climbing tour. However, CWI was performed early in the day (13:00–14:00), and sleep was monitored using wristwatch actigraphy, which does not allow sleep architecture assessment. In contrast, Robey et al. (2013) did not report any improvement in sleep architecture after the use of partial-body immersion at ≈20:15 following an intense cycling exercise compared with exercise alone.

To the best of our knowledge, no study has assessed the effect of whole-body CWI (head immersed) on sleep architecture and recovery in a sport setting. The aim of the present study was to investigate the effect of the depth of CWI (whole-body with head immersed vs. partial-body CWI) realized close to bedtime after high-intensity, intermittent running exercise on sleep architecture and recovery kinetics among well-trained runners. We hypothesized that a higher decline in *T*_core_ induced by whole-body immersion performed post-exercise would enhance SWS proportion and hasten the recovery process compared with a partial-body immersion.

## Materials and Methods

### Participants

Twelve well-trained male runners [mean ± *SD*; age = 28.0 ± 5.8 years; body mass = 65.7 ± 6.6 kg; height = 176.0 ± 8.6 cm; body fat estimated from the method of Durnin and Womersley (1974) = 9.8 ± 3.2%; maximal aerobic speed (MAS) = 18.1 ± 1.0 km·h^−1^; maximal oxygen uptake ($\dot{V}$O_2_max) = 66.0 ± 3.9 ml·min^−1^·kg^−1^] volunteered to participate. Participants were classified at level 4 according to the guidelines of De Pauw et al. (2013) for performance level classification in sport science research. All participants used to train three to six times a week with a required performance level <38 min per 10 km, $\dot{V}$O_2_max > 60 ml·min^−1^·kg^−1^ and MAS > 17 km·h^−1^. They were not accustomed to CWI and underwent a detailed medical history and examination by a medical doctor. This included an electrocardiogram at rest and an examination to exclude any contraindication to cold water exposure, e.g., cold hypersensitivity (Raynaud's phenomenon). The study was conducted according to the Declaration of Helsinki (1964: revised in 2001), and the protocol was approved by the local ethics committee (East III, France. Ref. 170605). The participants also provided their written informed consent before the initiation of experiments.

Actigraphy data (CamNtech, MotionWare 8) were collected for 3–15 days (BASELINE: 11.0 ± 7.2 days) before the start of the study and during the study to assess the sleep–wake patterns of each participant. They were instructed to sleep in the same home environment throughout the study and to respect the same bedtime and wake-up time (±30 min). No main effect (*p* > 0.05) of time, condition, and interaction between time and condition was noted for any actigraphic values from the two nights before experimentation to the end of each condition. The exclusion criteria checked prior to the start of the study were as follows: (a) an average sleep duration >9 or <6 h per night from Sunday to Thursday; (b) an average lights-out time earlier than 21:00 from Sunday to Thursday; (c) an average wake-up time later than 09:00 from Monday to Friday (Arnal et al., 2016); (d) a daily consumption of alcoholic beverages and/or more than 300 mg of caffeine per day and/or the use of antidepressant medications; (e) sleep complaints (i.e., Pittsburgh Sleep Quality Index >5; Buysse et al., 1989) and a non-extreme morning or evening chronotype on the Horne and Ostberg questionnaire (i.e., <31 and >69; Horne and Ostberg, 1976); (f) polysomnography-confirmed sleep disorders, such as sleep apnea (apnea–hypopnea index >10) and other sleep disorders (periodic limb movement syndrome, hypersomnia, insomnia, circadian sleep rhythm disorders, or narcolepsy); and (g) shift workers.

### Experimental Design

The design consisted of a familiarization session and three experimental conditions—a whole-body CWI including the head (WHOLE), a partial-body CWI up to the iliac crest (PARTIAL), and finally an out-of-water control condition (CONT)—all completed after a standardized simulated trail running (TRAIL). Two participants did not complete the CONT condition. The familiarization session was conducted at least 1 week before the experimental conditions and included the following: (a) a graded-exercise test; (b) a short period (≈5 min) of downhill and climb running on a treadmill, with the purpose of familiarization without inducing muscle damage; (c) familiarization with the protocol of lower limb muscle strength assessment corresponding to a maximal voluntary isometric contraction (MVIC) as well as countermovement jump (CMJ) test; (d) familiarization of WHOLE during ≈6 min; and (e) a night of familiarization with the polysomnography portable device in the same home environment as for the experimental nights to avoid the first night effect (Agnew et al., 1966).

In a randomized, crossover, counterbalanced order, participants performed WHOLE, PARTIAL, and, finally, CONT, all separated by a minimum of 1 week. The one-way ANOVA revealed no main effect of condition (*p* > 0.05) for the ambient temperature in the laboratory (23.5 ± 2.4°C) and relative humidity (44.1 ± 8.0%). Participants were asked to abstain from physical activity the day prior to and for the duration of each experimental condition. Food intake was standardized for all participants 3 days prior to and for the duration of each experimental condition. The meal plan was created by a nutritionist and included a variety of breads, cereals, milk/yogurts, meats, pasta/rice, fruit, and vegetables to ensure the adequate intake of macro- and micronutrients. Participants were hydrated regularly before and after the simulated trail but not during the trail. Additionally, they were not allowed to use any recovery strategy (e.g., compression garments, electrostimulation, massage, and stretching) during the protocol.

**Figure 1:**
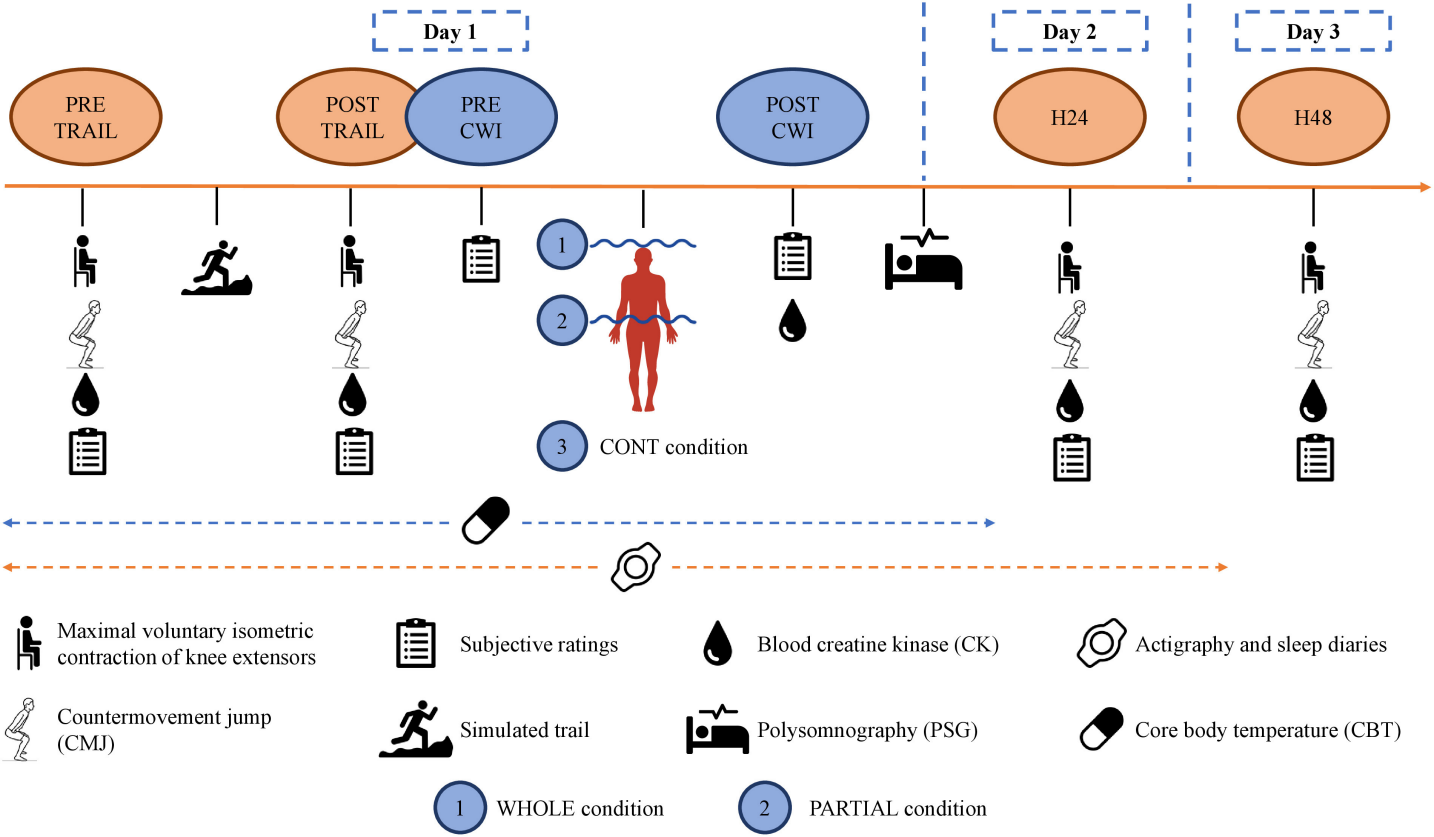
Study design.

In the three experimental conditions, participants arrived at ≈17:00 to start the testing battery (PRE-TRAIL) in the following order: subjective ratings, blood sampling, MVIC, and CMJ (Figure 1). They performed the simulated trail at ≈18:00. The test battery was repeated after (POST-TRAIL), and all participants were accompanied to the balneotherapy (approximately 250 m), drinking 500 ml of standardized milk beverage. They changed into their bathing suits and took a cold (≈15°C) shower during ≈2 min for sanitary purposes. They performed WHOLE, PARTIAL, or CONT at ≈19:55 and did not take a shower afterward. They ate a standardized meal at ≈20:15. Finally, the polysomnography equipment was set up (taking approximately 40 min) by a qualified practitioner, and participants went home to sleep in their usual environment. The one-way ANOVA revealed no main effect of condition (*p* > 0.05) for bedtime (23:25 ± 00:38), wake-up time (06:37 ± 00:49), and bedroom ambient temperature (22.1 ± 2.9°C) (iButtons, Embedded Data Systems, Lawrenceburg, Kentucky). Finally, participants returned to the laboratory 24 h (H24) and 48 h (H48) after the simulated trail at ≈17:00 to perform the same test battery.

### Graded-Exercise Test

The graded-exercise test was performed until exhaustion to determine MAS and $\dot{V}$O_2peak_ on a treadmill (Saturn 300/100r, h/p/cosmos Sports & Medical gmbh, Germany). The test started at 12 km·h^−1^ and increased 1 km·h^−1^ every 2 min with a slope at +1%. The last successful 2-min step was assigned to MAS and used to individualize the simulated trail intensity.

### Simulated Trail

For each experimental condition, participants completed a simulated trail adapted from Aloulou et al. (2019). The exercise lasted 48 min with five 9-min blocks and was shown to elicit a significant level of fatigue and muscle damage until 48 h after exercise (Aloulou et al., 2019). Heart rate (HR) was measured throughout the exercise. Each block included 4 min of downhill running (−12.5% gradient) followed by 3 min of flat running (0% gradient) and 2 min of uphill running (+10% gradient). During the downhill running, velocity was set to 80% of the MAS achieved during the graded-exercise test. The flat block included three 1-min runs at 100, 60, and 100% of MAS. During the uphill running, velocity was set to 65% of MAS.

### Cold Water Immersion Interventions

Following the POST-TRAIL testing battery, each participant completed 10 min of WHOLE, PARTIAL, or CONT. The whole-body immersion included the head and neck in a crouching position. Participants were fitted with a scuba kit and swimming pool glasses. For the partial-body immersion, participants were standing upright and immersed up to the iliac crest. The one-way ANOVA revealed no main effect of condition (*p* > 0.05) for water temperature (13.3 ± 0.2°C), in accordance with recommendations (Machado et al., 2016), and for the time to reach the required immersion level (03:23 ± 01:20 min). For CONT condition, participants sat down for 10 min in a controlled environment (19.2 ± 2.0°C; 47.9 ± 9.6% relative humidity).

### Measures

#### Core Body Temperature

Three hours before the simulated trail, participants ingested a radiotelemetry pill (BodyCap; e-Celsius^®^ Performance) to continuously record *T*_core_ with a frequency of 1 value per min. This method has been shown to be reliable and valid (Bongers et al., 2018), with an accuracy of 0.23°C, an intraclass correlation coefficient of 1.00, and a standard error of measurement of 0.03.

#### Sleep Recording and Analysis

##### Polysomnography

Polysomnography recordings were obtained the night after each CWI intervention using a portable device (Nox A1; Resmed). Polysomnography was performed following the technical specifications of the American Academy of Sleep Medicine manual for the scoring of sleep and associated events (Berry et al., 2017) including the following: six electroencephalography channels placed according to the international 10–20 electrodes placement system (F3-M2, F4-M1, C3-M2, C4-M1, O1-M2, and O2-M1); left and right electro-oculography; two chin electromyography channels, placed on the mentalis and submentalis; bilateral tibial electromyography; electrocardiography; and rib cage and abdominal wall motion via respiratory impedance. All data were scored using Noxturnal software version 5.1 (Resmed, USA) in 30-s epochs, according to the 2017 American Academy of Sleep Medicine (Berry et al., 2017). Each experimental night was analyzed by one trained specialist (>3,000 analyses in the last 10 years) who was blinded to the condition. Two distinct analyses were performed, one for the whole night and one for the first 180 min after sleep onset (Robey et al., 2013). The following dependent variables were calculated: total sleep time (min), the time spent in any stage of sleep (i.e., N1, N2, SWS, and REM); time in bed (min), the time between lights out getting up; wake after sleep onset (WASO; min), the time spent in bed awake minus sleep onset latency; sleep efficiency (%), total sleep time divided by time in bed ×100; sleep onset latency (min), the time between lights out and the first epoch of any stage of sleep (i.e., N1, N2, SWS, and REM); REM onset latency (min), the time between lights out and the first epoch of any stage of REM sleep stage; wake, N1, N2, SWS, REM, light (N1+N2), and NREM sleep (N1+N2+SWS) were defined as percentage of time spent compared with the time from sleep onset to waking up; arousal, a sudden change in electroencephalographic frequency—alpha, theta, and/or frequency >16 Hz—lasting at least 3 s and with the presence of at least 10 s of sleep before the change; and limb movement, an increase of ≥8 μV in the activity of the tibial electromyography lasting between 0.5 and 10 s. The arousal and limb movement indexes were defined as the number of counts per hour asleep.

##### The Spiegel Sleep Inventory

The Spiegel Sleep Inventory (SSI) was administered upon waking to assess the perceived sleep quality of each participant in each condition. The SSI is a self-administered questionnaire composed of six questions (score: 1–5) regarding sleep initiation, sleep quality and duration, nocturnal awakenings, dreams, and feeling refreshed in the morning. The global score is the sum of the six items. There are few data available on the psychometric validity of the SSI; however, it is a very simple and easy-to-use scale that is often used to assess the presence of insomnia (Léger et al., 2006).

#### Nocturnal Heart Rate

The ECG signal was derived from the right midclavicular and around 6 cm under the left armpit positions. The electrodes were connected to the polysomnographic system (Nox A1; Resmed, USA), and ECG data were continuously recorded at 200 Hz. Data were then converted into a European data format and imported into Kubios heart rate variability (HRV) software (version 3.3.1, 2019; MATLAB) for analysis. Nocturnal HRV and HR indexes were determined using the first 5-min stationary segment (free from arousals) in the first SWS sequence (determined via polysomnographic scoring) that lasted more than 15 min. Slow-wave sleep has been shown to better discriminate the state of sympathovagal balance than waking periods (Brandenberger et al., 2005). Electrocardiogram waveforms were analyzed to obtain temporal and frequency domain components. Time-domain variables, including mean R-R interval (RRi), the standard deviation of normal RRi (SDNN), and the root-mean square difference of successive normal RRi (RMSSD) were assessed. Frequency domains were assessed for the power densities in the low-frequency (LF; 0.04–0.15 Hz) and high-frequency (HF; 0.15–0.50 Hz) bands during each 5-min spectrum. The LF/HF ratios and the normalized LF/(LF+HF) ratios were then calculated.

#### Markers of Fatigue, Muscle Damage, and Subjective Assessments

##### Maximal Voluntary Isometric Contraction of Knee Extensors

Maximal voluntary isometric contraction of knee extensors was assessed at PRE-TRAIL, POST-TRAIL, H24, and H48 with a 90° knee angle and 80° hip angle using an isokinetic ergometer (Con-Trex Multi-Joint System) after a 10-min warm-up on a cyclo-ergometer at 100 W and 80 rpm. For each maximal contraction, the participants were instructed to extend their knees as fast and as hard as possible for 5 s, and all participants received standardized verbal encouragement from the same experimenter. Three MVICs of the knee extensor muscles were performed, with rest periods of 60 s. The best performance was defined as the highest peak force value of the three trials. The test–retest reliability was assessed by comparing the pre-values of the three experimental conditions. The typical error was 13.3 N·m (95% CI, 9.8 to 21.2 N·m), the intraclass correlation coefficient was 0.90 (95% CI, 0.72 to 0.97), and the coefficient of variation was 5.6%.

##### Countermovement Jump (CMJ) Performance

Countermovement jump (CMJ) performance was assessed at PRE-TRAIL, POST-TRAIL, H24, and H48 to evaluate neuromuscular fatigue and measured with photoelectric cells (Optojump^®^, Microgate, Bolzano, Italy) after a free joint warm-up. For each maximal effort, the participants were instructed to jump as high as possible while keeping hands on hips throughout the jump. The range of motion was free, and all participants received standardized verbal encouragement from the same experimenter. Three CMJs were performed, with rest periods of 60 s. The height of each jump was noted, and the best jump was defined as the highest jump of the three trials. The test–retest reliability was assessed by comparing the pre-values of the three experimental conditions. The typical error was 1.6 cm (95% CI, 1.2 to 2.5 cm), the intraclass correlation coefficient was 0.92 (95% CI, 0.79 to 0.98), and the coefficient of variation was 4.5%.

##### Blood Creatine Kinase [CK] Concentration

Blood creatine kinase [CK] concentration was assessed at PRE-TRAIL, POST-TRAIL, POST-CWI, H24, and H48 by collecting a 32-μl blood sample from a fingertip capillary puncture. The blood sample was then placed on a measurement strip and analyzed using a Reflotron Plus (Roche Diagnostics). The Reflotron Plus was calibrated according to the manufacturer's recommendations. The test–retest reliability was assessed by comparing the pre-values of the three experimental conditions. The typical error was 165.2 UI/L (95% CI, 125.4 to 262.7 UI/L), the intraclass correlation coefficient was −0.10 (95% CI, −0.44 to 0.39), and the coefficient of variation was 80.4%.

##### Rating of Perceived Exertion of the Session (RPE- S)

Rating of perceived exertion of the session (RPE-S) was assessed at POST-TRAIL. Participants answered the question “How was your workout?” using a scale from 0 (rest) to 10 (maximal; Foster, 1998).

##### The Well-Being Hooper Index

The well-being Hooper Index is a rating of general fatigue, stress, sleep perception, and muscle soreness (Hooper and Mackinnon, 1995). Participants were asked to subjectively evaluate at PRE-TRAIL, POST-TRAIL, POST-CWI, H24, and H48 for each condition the four items, using a 1–7 scale, with 1 representing the most positive rating and 7 representing the most negative rating, for each variable.

##### The Total Quality of Recovery (TQR) Scale

The total quality of recovery (TQR) scale was reported by the participants at POST-CWI, H24, and H48 for each condition. Scores varied from 6, “very, very poor recovery,” to 20, “very, very good recovery” (Kenttä and Hassmén, 1998).

##### Belief in the Anticipated Effectiveness

Belief in the anticipated effectiveness of WHOLE and PARTIAL was assessed at PRE-TRAIL and H48 for these conditions on a scale adapted from Broatch et al. (2014). Participants were instructed to choose on a 5-point Likert scale between two extremes points (1 indicating “strongly agree” and 5 indicating “strongly disagree”) by answering the following question: “Whole (or partial-) body cold water immersion allows a better recovery compared with passive recovery (no cold water immersion)?”

##### Perceived Thermal Comfort

Perceived thermal comfort (on a scale from −2 “very uncomfortable” to +2 “very comfortable”) and *sensation* (on a scale from −3 “cold” to +3 “hot”; Zhang and Zhao, 2008) were recorded for each participant at PRE-TRAIL, PRE-CWI, and POST-CWI.

### Statistical Analysis

Based on a previous study (Aloulou et al., 2019), we estimated that a sample size of 12 subjects would allow us to detect differences in MVIC after a simulated trail with power (1 – β) set at 0.80, a large effect size (>0.8) and an alpha of 0.05. *Post-hoc* power analysis revealed that this study was adequately powered, with actual power (1 – β) > 0.90 (G^*^Power program version 3.1.9.7). Statistical analyses were performed with the R program (version 1.4.869). Prior to the analysis, the Shapiro–Wilk test and the Mauchly test were employed to test the normality of the data and sphericity assumption, respectively. The Greenhouse–Geisser correction was conducted when the sphericity was violated to adjust the significance of the *F* ratios. Core body temperature, nocturnal HR, and markers of fatigue and exercise-induced muscle damage in WHOLE, PARTIAL, and CONT conditions were analyzed using a two-way (condition × time) repeated-measures analysis of variance (ANOVA). A log transformation was applied to the non-normalized data (i.e., MVIC, [CK], general fatigue, stress, muscle soreness, TQR, belief in the anticipated effectiveness of intervention, perceived thermal comfort, and sensation) to reduce non-uniformity bias. For *T*_core_ kinetics, 13-time points were used from the start to 120 min after the start of CWI intervention with 10-min intervals. For nocturnal *T*_core_, 13-time points were used from bedtime to 06:00 after bedtime with 30-min intervals. For polysomnography and HRV analysis, one-way ANOVAs with repeated measures were performed. The Friedman non-parametric test was performed for non-normalized data to observe the main effect of the experimental condition. Partial eta squared (η^2^_*p*_) is provided as measures of effect size for the two- and one-way ANOVA for repeated-measures, and Kendall's W value is provided for the Friedman test. When a significant main effect was found, Tukey honestly significant difference (HSD) *post-hoc* test or the non-parametric Conover test was performed. Effect sizes were calculated to interpret the magnitude of the mean difference between conditions with *d* < 0.2, *d* = 0.2–0.5, *d* = 0.5–0.8, and *d* > 0.8 considered trivial, small, moderate, and large, respectively (Cohen, 1988). Correlations between dependent variables were analyzed using the Pearson product-moment correlation coefficient (*r*). Results are expressed as the mean ± standard deviation (*SD*), and the level of significance was set at *p* < 0.05.

## Results

The one-way ANOVA revealed no main effect of condition (*p* > 0.05) for the mean HR during the simulated trail and RPE-S. Independent of the experimental condition, mean HR during the simulated trail was 83.5 ± 3.7% of maximal HR, and RPE-S was 7.8 ± 1.8 AU. A main effect of time (*p* < 0.001; η^2^_*p*_ = 0.92) for *T*_core_ throughout the simulated trail was found. Independent of the experimental conditions, *T*_core_ was 36.99 ± 0.55°C at the beginning of the simulated trail. At the end of the simulated trail, *T*_core_ significantly increased (*p* < 0.001; *d* = 2.75) with a large effect compared with PRE-TRAIL (38.94 ± 0.53°C).

### Effects of Cold Water Immersion Interventions on Thermal Responses, Sleep, and Heart Rate Variability

#### Core Body Temperature

**Figure 2:**
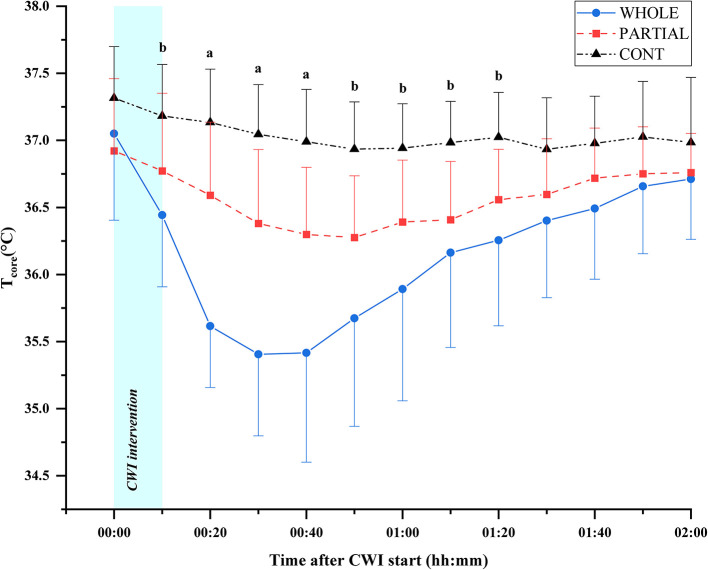
Core body temperature responses (°C) in WHOLE (*n* = 12), PARTIAL (*n* = 11), and CONT (*n* = 9). All data are presented as mean ± *SD*. ^a^WHOLE condition different to both PARTIAL and CONT conditions (*p* < 0.05). ^b^WHOLE condition different to CONT condition (*p* < 0.05).

Significant main effects of condition (*p* < 0.001; η^2^_*p*_ = 0.68), time (*p* < 0.001; η^2^_*p*_ = 0.68), and the interaction between condition and time (*p* < 0.001; η^2^_*p*_ = 0.53) were observed for *T*_core_ responses (Figure 2). *Post-hoc* analysis revealed a significantly lower *T*_core_ induced by WHOLE than CONT from the end of CWI intervention to 80 min after the start of CWI intervention with large effects (*p* < 0.05; *d* = 0.96–2.45). Compared with PARTIAL, WHOLE induced a significantly lower *T*_core_ from 20 to 40 min after the start of CWI intervention with large effects (*p* < 0.001; *d* = 1.61–2.04). During the whole night, the two-way ANOVA revealed no significant interaction between condition and time (*p* = 0.64; η^2^_*p*_ = 0.13). However, there was a significant main effect of condition (*p* < 0.05) for mean *T*_core_ (Table 1). Mean *T*_core_ was significantly higher in WHOLE (*p* < 0.01; *d* = 0.82) and PARTIAL (*p* < 0.05; *d* = 0.86) with a large effect compared with CONT condition.

**Table 1:** Polysomnography analysis, mean nocturnal heart rate, and *T*_core_ during the whole night in WHOLE (*n* = 12), PARTIAL (*n* = 12), and CONT (*n* = 10) conditions.

**Condition**	**WHOLE**	**PARTIAL**	**CONT**	***p*-Value**	**η^**2**^*_***p***_*/W**
Total sleep time (min)	398.4 ± 84.8	393.5 ± 57.7	389.6 ± 47.6	0.61	0.05
Time in bed (min)	447.5 ± 65.8	435.0 ± 54.5	441.7 ± 51.4	0.38	0.10
WASO (min)	26.4 ± 19.1	23.6 ± 16.5	37.3 ± 29.4	0.10	0.23
Sleep efficiency (%)	88.4 ± 9.2	90.5 ± 6.3	88.3 ± 6.6	0.50	0.07
Sleep latency (min)	8.7 ± 4.8	10.2 ± 9.8	13.8 ± 12.9	0.45	0.08
REM latency (min)	114.9 ± 44.6	98.2 ± 40.1	105.8 ± 38.8	0.55	0.06
Wake (%)	6.5 ± 5.1	5.6 ± 4.1	8.7 ± 6.5	0.12	0.21
Stage N1 sleep (%)	6.9 ± 2.2^a^	7.8 ± 2.4	8.6 ± 2.2	<0.01	0.41
Stage N2 sleep (%)	45.7 ± 5.9	47.3 ± 7.1	44.6 ± 6.4	0.07	0.25
SWS (%)	24.3 ± 5.9	22.1 ± 5.8	21.8 ± 4.0	0.33	0.12
NREM (%)	77.4 ± 5.5	77.2 ± 5.0	75.0 ± 6.3	0.74	0.03
REM (%)	16.5 ± 3.3	17.2 ± 5.1	16.3 ± 3.0	0.90	0.01
Light sleep (%)	52.7 ± 4.7	55.1 ± 6.9	53.2 ± 5.0	0.50	0.07
Arousals (/h)	9.2 ± 2.5^a^	9.4 ± 2.8^a^	12.4 ± 3.1	<0.01	0.49
Limb movements (/h)	5.2 ± 2.0^a^	8.5 ± 5.4	11.5 ± 6.5	<0.001	0.61
Mean heart rate (bpm)	50.2 ± 7.8	49.0 ± 6.5	49.3 ± 7.6	0.67	0.04
Mean *T*_core_ (°C)	36.47 ± 0.15^a^	36.43 ± 0.19^a^	36.27 ± 0.26	<0.05	0.43

*Data are presented as mean ± SD. WASO, wake after sleep onset; SWS, slow-wave sleep; NREM, non-rapid eye movement; REM, rapid eye movement*.

a *Significantly different from CONT condition (p < 0.05)*.

#### Sensation and Thermal Comfort

There was a significant interaction between condition and time for sensation (*p* < 0.01; η^2^_*p*_ = 0.42) and thermal comfort (*p* < 0.01; η^2^_*p*_ = 0.41). Immediately after WHOLE, large significantly lower sensation and thermal comfort were observed compared with both CONT and PARTIAL (*p* < 0.001; *d* = −2.39 to −1.37).

#### Polysomnography Analysis During the Whole Night

There was a main effect between conditions (*p* < 0.05) for N1 sleep, arousals, limb movements, and mean *T*_core_ (Table 1). *Post-hoc* analysis revealed a large significantly lower proportion of N1 sleep in WHOLE than CONT condition (*p* < 0.01; *d* = −1.73). The number of arousals was significantly higher in CONT than both WHOLE (*p* < 0.05; *d* = 1.01) and PARTIAL conditions (*p* < 0.05; *d* = 0.74). A large significant reduction in limb movements was noted in WHOLE compared with CONT condition (*p* < 0.01; *d* = −0.98). There were no main effects of condition on overall night polysomnographic sleep variables (Table 1).

#### Polysomnography Analysis During the First 180 Min After Sleep Onset

A significant main effect of condition (*p* < 0.05) was found for arousals and limb movements (Table 2). The number of arousals was significantly higher in CONT than both WHOLE (*p* < 0.05; *d* = 0.72) and PARTIAL conditions (*p* < 0.05; *d* = 0.55). Limb movements were significantly lower in WHOLE than both PARTIAL (*p* < 0.01; *d* = −0.62) and CONT conditions (*p* < 0.001; *d* = −0.92). A trend of a significant main effect of condition was found for SWS (*p* = 0.08) and REM sleep proportions (*p* = 0.09). Slow-wave sleep was moderately higher in WHOLE than PARTIAL (*p* < 0.05; *d* = 0.59). There was no main effect of condition on other polysomnographic sleep variables.

**Table 2:** Polysomnography analysis, mean nocturnal heart rate, and *T*_core_ during the first 180 min after sleep onset in WHOLE (*n* = 12), PARTIAL (*n* = 12), and CONT (*n* = 10) conditions.

**Condition**	**WHOLE**	**PARTIAL**	**CONT**	***p*-value**	**η^**2**^*_***p***_*/W**
Total sleep time (min)	172.2 ± 6.8	173.1 ± 5.3	162.5 ± 22.7	0.50	0.07
WASO (min)	7.7 ± 6.8	6.9 ± 5.2	17.4 ± 22.7	0.58	0.05
Wake (%)	4.3 ± 3.8	3.9 ± 2.9	9.7 ± 12.6	0.58	0.05
Stage N1 sleep (%)	6.9 ± 2.4	7.1 ± 2.5	7.8 ± 2.6	0.70	0.04
Stage N2 sleep (%)	41.9 ± 7.8	42.9 ± 7.8	40.6 ± 7.4	0.24	0.15
SWS (%)	39.7 ± 7.4^b^	34.6 ± 6.6	35.1 ± 10.1	0.08	0.25
NREM (%)	88.6 ± 6.3	84.6 ± 5.6	83.5 ± 13.7	0.41	0.09
REM (%)	7.2 ± 3.8	11.5 ± 5.2	6.8 ± 2.9	0.09	0.23
Light sleep (%)	48.8 ± 7.2	50.1 ± 9.1	48.4 ± 7.4	0.84	0.02
Arousals (/h)	10.6 ± 2.7^a^	10.5 ± 3.3^a^	13.2 ± 3.4	0.05	0.28
Limb movements (/h)	4.4 ± 2.1^a, b^	9.3 ± 9.0	11.4 ± 8.9	<0.01	0.57
Mean heart rate (bpm)	52.3 ± 8.6	50.4 ± 7.4	51.1 ± 8.7	0.53	0.07
Mean *T*_core_ (°C)	36.51 ± 0.19	36.46 ± 0.25	36.31 ± 0.29	0.07	0.28

*Data are presented as mean ± SD. WASO, wake after sleep onset; SWS, slow-wave sleep; NREM, non-rapid eye movement; REM, rapid eye movement*.

a *Significantly different from CONT condition (p < 0.05)*.

b *Significantly different from PARTIAL condition (p < 0.05)*.

#### Subjective Sleep Evaluation

A main effect of condition was found for the “feeling refreshed in the morning” item (*p* < 0.05). Compared with CONT, both WHOLE (*p* < 0.05; *d* = 0.82) and PARTIAL conditions (*p* < 0.05; *d* = 0.91) largely improved the “feeling refreshed in the morning” item. There was no main effect of condition on other SSI items.

#### Nocturnal Heart Rate

There was no significant main effect of condition on the mean HR during the whole night (Table 1). HRV analysis showed a significant main effect of condition on RMSSD, LF, and HF (*p* < 0.05; Table 3). *Post-hoc* analysis revealed a large significant reduction in RMSSD in WHOLE compared with both PARTIAL (*p* < 0.05; *d* = −0.83) and CONT (*p* < 0.01; *d* = −0.82). A large reduction in LF was noted in WHOLE compared with both PARTIAL (*p* < 0.01; *d* = −1.01) and CONT (*p* < 0.05; *d* = −1.01). After the WHOLE condition, a large significant reduction in HF was observed compared with both PARTIAL (*p* < 0.05; *d* = −0.89) and CONT (*p* < 0.01; *d* = −0.93). No main effect of condition was noted for RRi, HR, the normalized LF/(LF+HF) ratio, and the LF/HF ratio (*p* > 0.05). SDNN in WHOLE was moderately and largely lower than PARTIAL (*p* = 0.06; *d* = −0.71) and CONT (*p* < 0.05; *d* = −0.91), respectively.

**Table 3:** Heart rate variability analysis during the first SWS sequence in WHOLE (*n* = 12), PARTIAL (*n* = 12), and CONT (*n* = 10) conditions.

**Condition**	**WHOLE**	**PARTIAL**	**CONT**	***p*-Value**	**η^**2**^*_***p***_*/W**
RRi (ms)	1,150.5 ± 212.3	1,219.8 ± 241.5	1,217.9 ± 259.7	0.20	0.17
HR (bpm)	53.7 ± 9.4	50.8 ± 9.0	51.3 ± 10.9	0.41	0.09
SDNN (ms)	43.5 ± 20.5^a^	61.2 ± 36.9	60.7 ± 27.2	0.06	0.28
RMSSD (ms)	46.0 ± 27.6^a^^,^^b^	71.1 ± 51.0	70.1 ± 41.5	<0.05	0.39
LF (ms^2^)	911.3 ± 628.6^a^^,^^b^	2,386.4 ± 2,880.0	1,993.2 ± 1,707.1	<0.01	0.37
HF (ms^2^)	751.0 ± 764.7^a^^,^^b^	2,055.5 ± 2,538.9	1,748.7 ± 1,766.4	<0.05	0.37
LF/(LF+HF) (AU)	62.6 ± 16.8	59.2 ± 22.0	58.5 ± 17.2	0.61	0.05
LF/HF (AU)	2.6 ± 2.8	2.5 ± 2.8	2.0 ± 1.9	0.15	0.19

*Data are presented as mean ± SD*.

a *Significantly different from CONT condition (p < 0.05)*.

b *Significantly different from PARTIAL condition (p < 0.05)*.

### Effects of Simulated Trail and Between-Condition Differences on Recovery Kinetics

No main effects of condition and interaction between condition and time (*p* > 0.05) were observed for MVIC, CMJ, [CK], general fatigue, muscle soreness, and stress up to 48 h after simulated trail (Supplementary Material 1). There was no significant difference between conditions (*p* > 0.05) at PRE-TRAIL and POST-TRAIL for MVIC, CMJ, [CK], general fatigue, muscle soreness, and stress, suggesting similar fatigue and muscle damage after exercise in WHOLE, PARTIAL, and CONT conditions. However, a main effect of time (*p* < 0.05) was found for MVIC, CMJ, [CK], general fatigue, and muscle soreness. Independent of the experimental conditions, *post-hoc* analysis revealed a significant large and moderate decrease in MVIC at POST-TRAIL (*p* < 0.001; *d* = −1.17) and H24 (*p* < 0.01; *d* = −0.56) compared with PRE-TRAIL. There was a significantly lower CMJ at H24 than PRE-TRAIL with a large effect (*p* < 0.001; *d* = 0.93). An increase in [CK] from POST-TRAIL to H48 (*p* < 0.01; *d* = 0.67–2.16) was found compared with PRE-TRAIL. General fatigue was significantly higher at POST-TRAIL than PRE-TRAIL with a large effect (*p* < 0.001; *d* = 1.26). Muscle soreness was significantly higher than PRE-TRAIL from POST-TRAIL to H48 (*p* < 0.01; *d* = 0.64–1.52). There was a significant main effect of condition (*p* < 0.05) on TQR and belief in the effectiveness of CWI interventions. Irrespective of time assessment, TQR was significantly and moderately higher in both WHOLE (*p* < 0.01; *d* = 0.62) and PARTIAL (*p* < 0.01; *d* = 0.62) compared with CONT. A higher belief in the effectiveness of CWI interventions was found in both WHOLE (*p* < 0.001; *d* = 3.33) and PARTIAL (*p* < 0.001; *d* = 3.03) compared with CONT.

## Discussion

The aim of the present study was to investigate the effect of whole- (head immersed) and partial-body CWI after a high-intensity, intermittent running exercise on sleep architecture and recovery kinetics among well-trained athletes. The primary results showed the following: (a) both WHOLE and PARTIAL induced a significant decrease in arousals compared with CONT, while only WHOLE decreased limb movements compared with CONT; (b) WHOLE induced a significant reduction in both sympathetic and parasympathetic modulation compared with both PARTIAL and CONT; and (c) no significant differences were observed in markers of fatigue and exercise-induced muscle damage recovery between conditions.

Irrespective of the experimental conditions, the mean HR during the simulated trail was 83.5 ± 3.7% of maximal HR, whereas RPE-S achieved 7.8 ± 1.8 AU. In addition, the simulated trail induced a significant decrease in MVIC (−6.2%) and CMJ (−5.6%) 24 h after the simulated trail, whereas [CK] (+57.5%) and muscle soreness (+1.9 AU) were still significantly higher 48 h after exercise. These results suggest that the simulated trail induced a high level of metabolic and neuromuscular fatigue with the presence of mild muscle damage (Paulsen et al., 2012). To the authors' knowledge, the present study is the first to compare the effects of WHOLE and PARTIAL CWI on sleep architecture and recovery in a sport setting. The present results showed that WHOLE induced a significantly lower *T*_core_ compared with CONT up to 80 min after the start of immersion. This result is consistent with previous studies (Zhang et al., 2015; Stephens et al., 2017), which highlighted the need to expose an important body surface in cold water to enhance convective heat dissipation and decrease *T*_core_. Additionally, the immersion of the head in the present study may have contributed to a larger decline in *T*_core_ compared with partial-body immersion. Pretorius et al. (2006) showed that immersing the head in cold water (17°C) increases the rate of *T*_core_ decline (≈42%) compared with keeping the head above the water surface. A redistribution of blood flow to the face and scalp in response to stimulation of thermosensitive and/or trigeminal receptors as well as peripheral vasoconstriction may be potentially involved mechanisms (Pretorius et al., 2006).

We hypothesized that a higher decline in *T*_core_ induced by WHOLE would enhance SWS proportion during the subsequent night. Our hypothesis was only partially confirmed. To our knowledge, only one study previously used polysomnography to examine the effect of CWI following exercise on sleep quantity and quality among well-trained endurance athletes (Robey et al., 2013). Accordingly, Robey et al. (2013) reported no additional benefits procured by PARTIAL CWI compared with no CWI following an intense cycling exercise on sleep architecture, despite the fact that PARTIAL CWI induced a decrease in *T*_core_ during 90 min after bedtime. The maximal rate of decline in *T*_core_ close to bedtime—characterized by the distal-to-proximal skin temperature gradient and heat dissipation—has been associated with an increased sleep propensity (Berger and Phillips, 1995; Kräuchi and Deboer, 2010). In the present study, a significant increase in SWS proportion (+5.1%) during the first 180 min after sleep onset in WHOLE compared with PARTIAL was observed. The first part of the night is characterized by a high proportion of SWS (Akerstedt and Nilsson, 2003), which is involved in important neurophysiological functions such as growth hormone release, immunity, and cleaning of metabolites (Léger et al., 2018). Consequently, this sleep period may play an important part in the athletes' recovery process. The absence of a main effect of condition on overall night SWS proportion may be explained by the high mean SWS proportion during the CONT condition compared with the normal population (21.8 vs. 16%; Ohayon et al., 2004). As a consequence, a ceiling or maximum level of SWS in a single night (Taylor et al., 1997) beyond which even WHOLE could not induce an additional increase in SWS is possible. Finally, WHOLE appears as a promising strategy to increase SWS proportion during the first part of the night, which are crucial in the athlete's recovery process after training or a competition (Halson and Juliff, 2017; Walsh et al., 2020).

In addition to the positive effect of WHOLE on SWS proportion during the first part of the night, WHOLE and PARTIAL decreased arousals during the whole night compared with CONT. An arousal is defined by the National Sleep Foundation as an abrupt change in activity, which may cause a change in sleep stage from a deep stage of NREM sleep to a light stage, or from REM sleep toward wakefulness, with the possibility of awakening as the final outcome (Ohayon et al., 2017); and it is characteristic of sleep fragmentation (Ekstedt et al., 2004). The origin of an arousal is usually cortical, but it can be generated in response to sensory perturbations such as abnormal movement during sleep (Picchietti and Winkelman, 2005; Tuomilehto et al., 2017). This is supported in the present study by a significant correlation between arousals and limb movements (*r* = 0.43; *p* < 0.05; *n* = 34). Previous studies suggested that overreached athletes are more active during sleep compared with a phase of normal or reduced training load (Taylor et al., 1997; Hausswirth et al., 2014). Some authors proposed that higher muscle fatigue and muscle soreness during intensified training periods promote movements during sleep in an attempt to stay comfortable (Taylor et al., 1997; Sargent et al., 2016). Our results showed that WHOLE induced a large significant reduction in limb movements compared with CONT during the whole night, which may be a relevant recovery strategy to reduce limb movements and favoring sleep continuity. However, WHOLE did not alleviate muscle soreness in the present study, and its responsibility in limb movement reduction cannot be confirmed. Minett et al. (2014) showed a reduction in prefrontal cortex oxygenation following 20 min CWI up to mesosternal (10°C), which may alter the central motor output (Amann and Kayser, 2009). Future studies are required to ascertain the effects of WHOLE on the central nervous system and limb movement reduction during the night.

In the present study, WHOLE induced a significant decrease in RMSSD and HF compared with PARTIAL and CONT during the first SWS sequence of the night. A marked SDNN reduction was found in WHOLE compared with CONT. These results suggest that WHOLE decreased parasympathetic modulation. The present results differ from those reported elsewhere, which notably demonstrated an increase in parasympathetic activity immediately (Buchheit et al., 2009; Stanley et al., 2013) and the morning after (Al Haddad et al., 2012) PARTIAL CWI. The HRV analysis was presently performed during SWS, which offers a self-controlled and quiet moment of HRV observation (Brandenberger et al., 2005), making comparisons with the previously mentioned studies difficult. However, WHOLE probably induced important thermal stress and sympathetic hyperactivity (Datta and Tipton, 2006), which may explain a decrease in parasympathetic modulation during SWS compared with PARTIAL and CONT conditions. Cardiac parasympathetic activity during recovery from exercise may be indicative of an athlete's readiness to perform a high-intensity exercise the following day (Stanley et al., 2013). In the present study, WHOLE-induced parasympathetic activity decrease may be potentially deleterious for elite athletes in the context of consecutive high performance achievement (Hynynen et al., 2006). WHOLE induced a significant decrease in LF compared with PARTIAL and CONT. However, it has been suggested that LF does not reflect sympathetic nerve activity, whereas the measure of baroreflex function using LF is still being debated (Goldstein et al., 2011; Martelli et al., 2014). Additionally, both thermal sensation and comfort after WHOLE were altered compared with PARTIAL and CONT, which may be a potential barrier to implement WHOLE on the field in addition to the logistics constraints inherent to bathing the entire body (Bishop, 2008). Future studies are required to assess cooling strategies inducing a lesser extent of thermal stress while improving sleep architecture, e.g., head-only CWI.

Based on previous findings (Wilcock et al., 2006; Poppendieck et al., 2013; Stephens et al., 2017), we hypothesized that a higher decline in *T*_core_ induced by CWI interventions may hasten exercise-induced muscle damage recovery. Our hypothesis was rejected since WHOLE and PARTIAL procured no additional benefits on MVIC, CMJ, [CK], muscle soreness, fatigue general, and stress compared with CONT throughout the 48-h recovery period. These results are consistent with previous studies that reported a trivial/small with moderate-to-high heterogeneity effect induced by CWI on strength, jump performance recovery, and muscle soreness (Leeder et al., 2012; Poppendieck et al., 2013). In addition, we noted a more positive belief in the effectiveness of WHOLE and PARTIAL compared with CONT. Broatch et al. (2014) reported that CWI-induced performance recovery may be at least partially related to a placebo effect, highlighting the importance to encourage athletes' belief in order to improve subjective ratings and performance recovery. Future studies are required to assess the effect of daily cooling strategies on the consecutive night's sleep architecture and recovery kinetics.

## Limitations

Due to practical issues, sleep was monitored using polysomnography only during the night after exercising, and markers of fatigue/muscle damage were not collected beyond 48 h after the simulated trail. Future studies are consequently required to assess the effect of WHOLE on the consecutive night's sleep architecture and recovery kinetics beyond 48 h after exercise. Finally, participants performed the out-of-water condition as a control condition. The absence of a thermoneutral water immersion condition (i.e., hydrostatic pressure without cooling) prevents any conclusion on the potential effect of hydrostatic pressure on sleep and recovery kinetics.

## Conclusion

The present study showed that WHOLE and PARTIAL CWI performed after a high-intensity, intermittent running exercise largely decreased sleep arousals compared with CONT, while WHOLE only decreased limb movements compared with CONT. This study contributes to the development of strategies to enhance sleep and recovery for elite athletes who are exposed to periods of intense training/competition and disturbed sleep (Gupta et al., 2017). Our results suggest that WHOLE may be particularly useful for athletes to reduce limb movements and sleep arousals after exercise. However, WHOLE and PARTIAL did not hasten the recovery process compared with CONT during the 48-h follow-up period, and WHOLE decreased parasympathetic activity. Furthermore, the use of WHOLE by athletes on the field may be hampered by poor sensation and thermal comfort. Future studies should be conducted to explore the potential benefits of solely cooling the head—inducing a lesser extent of thermal stress—to increase sleep propensity during a training and competition period.

## Data Availability Statement

The raw data supporting the conclusions of this article will be made available by the authors, without undue reservation.

## Ethics Statement

The studies involving human participants were reviewed and approved by the local ethics committee (East III, France. Ref. 170605). The patients/participants provided their written informed consent to participate in this study.

## Author Contributions

MC and MN conceived and designed the research and analyzed and interpreted data. MC conducted experiments, wrote the first draft of the manuscript, and contributed to the writing of the final paper. FP, VG, and AA contributed to the data collection. All authors contributed to the article and approved the submitted version.

## Conflict of Interest

The authors declare that the research was conducted in the absence of any commercial or financial relationships that could be construed as a potential conflict of interest.
