# Supplementary material for: Effect of the Depth of Cold Water Immersion on Sleep Architecture and Recovery Among Well-Trained Male Endurance Runners
Source: Front Sports Act Living. 2021 Mar 31;3:659990. doi: 10.3389/fspor.2021.659990 (PMC8044518; doi:10.3389/fspor.2021.659990)
Supplement: Supplementary file 1 [file Table_1.DOCX]

**Supplementary Material 1**: Differences between WHOLE (n = 12), PARTIAL (n = 12) and CONT (n = 10) conditions on markers of fatigue and exercise-induced muscle damage throughout the 48-h recovery period.

|  |  | **MVIC (N.m)** | **CMJ (cm)** | **[CK] (UI/L)** | **General fatigue (AU)** | **Muscle soreness (AU)** | **Stress (AU)** | **TQR (AU)** | **Belief in CWI interventions (AU)** |
| --- | --- | --- | --- | --- | --- | --- | --- | --- | --- |
| **Main effect** | *Condition* | p = 0.81  *ƞ^2^p* = 0.03 | p = 0.25  *ƞ^2^p* = 0.16 | p = 0.86  *ƞ^2^p* = 0.02 | p = 0.84  *ƞ^2^p* = 0.02 | p = 0.18  *ƞ^2^p* = 0.18 | p = 0.54  *ƞ^2^p* = 0.07 | p < 0.05  *ƞ^2^p* = 0.29 | p < 0.001  *ƞ^2^p* = 0.81 |
|  | *Time* | p < 0.001  *ƞ^2^p* = 0.61 | p < 0.05  *ƞ^2^p* = 0.34 | p < 0.001  *ƞ^2^p* = 0.73 | p < 0.001  *ƞ^2^p* = 0.60 | p < 0.001  *ƞ^2^p* = 0.67 | p = 0.15  *ƞ^2^p* = 0.17 | p = 0.83  *ƞ^2^p* = 0.01 | p = 0.31  *ƞ^2^p* = 0.11 |
|  | *Condition*  *x Time* | p = 0.37  *ƞ^2^p* = 0.12 | p = 0.77  *ƞ^2^p* = 0.07 | p = 0.47  *ƞ^2^p* = 0.10 | p = 0.26  *ƞ^2^p* = 0.13 | p = 0.18  *ƞ^2^p* = 0.17 | p = 0.19  *ƞ^2^p* = 0.14 | p = 0.59  *ƞ^2^p* = 0.06 | p = 0.10  *ƞ^2^p* = 0.23 |
| **PRE TRAIL** | WHOLE | 215.5 ± 26.5 | 34.0 ± 5.4 | 262.6 ± 150.0 | 3.5 ± 1.2 | 2.7 ± 0.9 | 2.5 ± 1.0 |  | 1.8 ± 0.7 |
|  | PARTIAL | 227.8 ± 44.0 | 33.9 ± 5.4 | 258.2 ± 205.8 | 3.3 ± 1.3 | 2.2 ± 1.2 | 3.0 ± 1.7 |  | 1.9 ± 0.8 |
|  | CONT | 230.8 ± 42.5 | 34.4 ± 4.5 | 209.7 ± 83.2 | 2.8 ± 0.9 | 2.0 ± 0.7 | 2.3 ± 0.9 |  | Reference |
|  |  |  |  |  |  |  |  |  |  |
| **POST TRAIL** | WHOLE | 195.8 ± 28.7 | 33.0 ± 5.0 | 370.5 ± 223.4 | 5.0 ± 0.7 | 4.4 ± 1.3 | 2.6 ± 1.0 |  |  |
|  | PARTIAL | 214.1 ± 40.9 | 33.5 ± 5.3 | 373.8 ± 279.1 | 5.2 ± 1.5 | 4.4 ± 0.7 | 2.7 ± 1.2 |  |  |
|  | CONT | 206.0 ± 43.2 | 33.4 ± 4.3 | 318.9 ± 103.8 | 4.7 ± 0.5 | 4.2 ± 1.5 | 2.3 ± 0.8 |  |  |
|  |  |  |  |  |  |  |  |  |  |
| **POST CWI** | WHOLE |  |  | 447.6 ± 216.2 | 3.5 ± 1.2 | 3.2 ± 1.3 | 2.3 ± 1.4 | 14.4 ± 2.2 |  |
|  | PARTIAL |  |  | 413.5 ± 220.0 | 3.5 ± 1.0 | 3.0 ± 1.4 | 1.8 ± 0.8 | 15.2 ± 1.2 |  |
|  | CONT |  |  | 367.5 ± 112.4 | 3.6 ± 1.1 | 3.7 ± 1.2 | 1.8 ± 0.9 | 13.6 ± 3.3 |  |
|  |  |  |  |  |  |  |  |  |  |
| **H24** | WHOLE | 203.6 ± 38.3 | 32.0 ± 5.4 | 730.2 ± 408.0 | 3.3 ± 1.1 | 4.0 ± 1.3 | 1.8 ± 1.0 | 15.0 ± 2.7 |  |
|  | PARTIAL | 212.7 ± 42.1 | 32.3 ± 4.9 | 580.3 ± 395.4 | 3.4 ± 1.4 | 4.0 ± 1.3 | 2.3 ± 1.2 | 14.5 ± 2.4 |  |
|  | CONT | 215.8 ± 66.8 | 32.3 ± 5.0 | 607.1 ± 281.9 | 4.2 ± 0.8 | 4.8 ± 0.8 | 2.4 ± 1.2 | 13.1 ± 3.0 |  |
|  |  |  |  |  |  |  |  |  |  |
| **H48** | WHOLE | 226.1 ± 43.4 | 33.3 ± 6.2 | 405.3 ± 214.4 | 2.8 ± 0.8 | 3.9 ± 1.2 | 2.1 ± 1.0 | 15.3 ± 2.3 | 1.9 ± 0.9 |
|  | PARTIAL | 222.3 ± 43.6 | 33.2 ± 5.2 | 369.5 ± 162.7 | 3.3 ± 1.1 | 3.8 ± 1.6 | 2.3 ± 1.4 | 14.9 ± 2.8 | 2.3 ± 1.2 |
|  | CONT | 218.0 ± 53.6 | 33.8 ± 5.4 | 384.8 ± 209.3 | 3.3 ± 1.3 | 4.9 ± 1.0 | 1.9 ± 1.0 | 12.8 ± 3.6 | Reference |

Data are presented as mean ± SD. MVIC: maximal voluntary isometric contraction; CMJ: countermovement jump; [CK]: creatine kinase levels; TQR: Total quality of recovery.
